# Oral Antineoplastic Agents: Assessing the Delay in Care

**DOI:** 10.1155/2015/512016

**Published:** 2015-10-28

**Authors:** Brandi Anders, Alexandra Shillingburg, Michael Newton

**Affiliations:** ^1^Wake Forest Baptist Health, Medical Center Boulevard, Winston-Salem, NC 27157, USA; ^2^West Virginia University Healthcare, 1 Medical Center Drive, Morgantown, WV 26506, USA; ^3^Clinical Department, West Virginia University School of Pharmacy, Morgantown, WV 26506, USA

## Abstract

The study was undertaken to determine the length of time between when a prescription for an oral antineoplastic agent is written by the provider and when the medication is received by the patient and to identify risk factors that significantly increase time to medication receipt. First-time fill prescriptions for oral antineoplastic agents were identified. The date the prescription was written and received by the patient was determined. A retrospective review was completed to gather additional information, including prescribed medication, indication, insurance coverage, patient assistance program use, dispensing pharmacy, and prior authorization requirements. The data was analyzed through multivariate statistical analysis and used to identify risk factors that may significantly increase the time to medication receipt. A total of 58 patients were included in the study. A median of 8 days elapsed between when the medication was prescribed and when it was received by the patient. Medication prescribed, absence of a Risk Evaluation Mitigation Strategies (REMS) program, and insurance type are factors that increased time to medication receipt. An understanding of the median time involved, as well as factors affecting the time to delivery of prescriptions, will help healthcare providers better plan and prepare for the use of oral antineoplastic agents.

## 1. Background

For the past several decades, cancer treatment has entailed primarily intravenous delivery of antineoplastic agents. Hospital services, as well as outpatient oncology infusion centers, have been organized around this type of medication administration. However, in recent years, the use of oral antineoplastic agents has steadily increased among patients with a cancer diagnosis. Currently, there are more than sixty oral antineoplastic drugs available, 22 of which are oral kinase inhibitors that have gained worldwide approval since 2001 [1]. Experts now estimate that more than one-quarter of the 400 antineoplastic agents now under development are planned as oral drugs [2–4]. In 2013, 5 of 8 newly approved cancer therapies were in an oral formulation [5, 6]. Several surveys have shown that most patients prefer oral antineoplastic drugs to intravenous treatment primarily for the convenience of a home-based therapy and ease of use [2, 3]. The use of oral antineoplastic agents for cancer treatment removes the routine and continuous monitoring that was included with intravenous treatment. With the increasing use of oral agents, patients now have more responsibility for monitoring and reporting side effects to their health care providers [3, 7].

**Figure 1:**
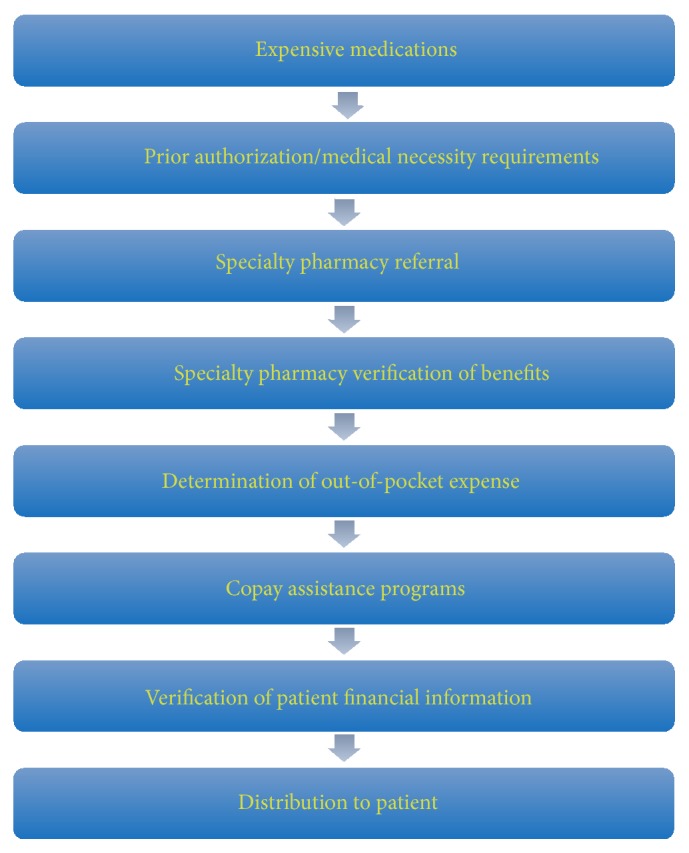
Process for obtaining medication.

While they have the added benefit of convenience, most new oral antineoplastic agents are more expensive than traditional intravenous chemotherapy. They are typically billed to the patient's prescription drug insurance rather than through their general medical coverage as with intravenously administered therapies [3, 8]. The medications also generally require the use of a specialty pharmacy that must mail or deliver these medications to the patients' homes [7]. Due to the increased costs associated with oral antineoplastic agents, many pharmacy benefit plans have implemented cost-containment mechanisms [3, 7]. This can include the use of prior authorization or medical necessity requirements, causing a delay in therapy initiation [7]. This can also result in increased expense to the patient, due to placement of medications in higher copayment tiers [3, 7]. After the prior authorization or medical necessity requirements have been met, the prescription is referred to a specialty pharmacy. The specialty pharmacy will verify the patient's insurance information and determine the out-of-pocket cost of the medication. Patient's insurance copays typically average between 10% and 20% of the drug cost. With the average cost of a new oral antineoplastic drug in 2012 approximating $10,000, paying for medications can be a significant out-of-pocket expense and burden for patients. To help alleviate these out-of-pocket expenses, patients who are uninsured or underinsured can often participate in patient assistance programs offered by drug manufacturers or other agencies. The paperwork required for participation in these programs is complex and time-consuming, often requiring detailed income information. All of these factors contribute to a delay in drug therapy initiation (Figure 1).

The current use of oral antineoplastic agents in the treatment of cancer has drastically increased over the past few years. Patient access to these agents is difficult, often leading to delays in the initiation of therapy. Since the consequences of delaying the initiation of cancer therapy can be severe, it is crucially important to have a complete understanding of the time involved and barriers associated with delayed start of treatment.

## 2. Methods

This study was a retrospective observational study which evaluated the amount of time (in days) that elapsed between when a prescription for an oral antineoplastic agent was written and when it was received by the patient. The study also sought to identify factors that could increase time to medication receipt. Institutional review board approval was obtained prior to data collection.

### 2.1. Study Patients

Patients 18 years of age and older, who received a new prescription (first-time fill) for an oral antineoplastic agent, were included in the study. Patients were excluded from the study if they were less than 18 years of age, participating in a clinical trial where the medication was provided by the study sponsor or receiving a prescription for a hormonal therapy agent.

### 2.2. Data Collection

Social workers and nurse clinicians from the cancer center assisted by compiling a list of patients receiving a prescription for a first-time fill of an oral antineoplastic agent. The date the prescription was written and received by the patient was obtained by retrospective review of the patient's electronic medical record and progress notes. In cases where the date of medication receipt was not outlined in patient progress notes, the date was obtained by directly calling the specialty pharmacy providing the medication. A retrospective chart review was completed to gather additional information used to identify risk factors that could significantly increase the time to medication receipt. Data collected included prescribed medication, demographic data (age and gender), medication indication, insurance coverage, usage of a patient assistance program, dispensing pharmacy, reasons for not filling prescription (if applicable), and if the prescription required a prior authorization. It was then denoted whether each medication required the use of a Risk Evaluation Mitigation Strategies (REMS) program.

The primary outcome was defined as the number of days that elapsed from the date the prescription was written to the date the prescription was received by the patient. Secondary outcomes included review of demographic data, medication prescribed, presence of a REMS component, type of primary insurance, presence of secondary insurance program, out-of-pocket cost of medication, patient assistance program use, and prior authorization requirements to determine which factors significantly increased time to medication receipt.

### 2.3. Statistics

Descriptive statistics and exploratory data analysis were preformed to summarize patient characteristics. Categorical data is described using contingency tables. Continuously scaled measures are summarized with descriptive statistical measures including mean (±SD). Wilcoxon rank sum test and Kruskal-Wallis test were used to examine the difference of time to medication receipt among different subgroups. A *p* value < 0.05 was considered statistically significant. Statistical analyses were carried out using SAS 9.1 (SAS Institute, Cary, NC) and S-Plus, version 7.0 (Insightful Corp., Seattle, WA) software.

## 3. Results

A total of 58 patients were identified and included in the study. The study population was well balanced with respect to gender and age, with a median age of 60.5 years (Table 1). Additional information analyzed can be seen in Table 2. There was a mean of eight days between when the prescription was written and when it was received by the patient. Patient age, gender, use of secondary insurance, out-of-pocket medication cost to patient, use of patient assistance program, and prior authorization requirements did not affect time to medication receipt. The time to receipt of medication was significantly affected by the medication prescribed, presence or absence of REMS requirement, and the type of insurance used (Table 2).

The most common medications prescribed were capecitabine, temozolomide, pomalidomide, and lenalidomide. Capecitabine required the longest amount of days to medication receipt (14.06 ± 12.31 days). Prescriptions for lenalidomide required the least amount of time to medication receipt (4.83 ± 2.32 days). Medication prescribed had a significant impact on time to receipt (*p* = 0.03). Medications with a REMS program required significantly less time to receipt than those without a REMS requirement (6.07 ± 3.56 days with requirement versus 10.42 ± 8.71 days without requirement; *p* = 0.02). Type of insurance used also had a significant impact on time to medication receipt. Patients with Medicare A&B required the longest amount of time to receive the medication (13.09 ± 13.66 days), and those with Medicare D required the least amount of time (5.67 ± 3.15 days). The difference in time to medication receipt with regard to insurance coverage was statistically significant (*p* = 0.003). As there was only one patient in the study with no insurance, the *p* value was calculated by excluding this patient.

## 4. Discussion

The results of this study showed the type of medication prescribed had a significant impact on time to medication receipt. All of the various clinics at the cancer center were included in this review, which are comprised of both solid tumor (breast cancer, lung cancer, head and neck cancer, etc.) and hematologic malignancy (leukemia, lymphoma, etc.) clinics. As is common in cancer therapy, certain medications are used more commonly or exclusively in one specific disease state. As such, these medications are prescribed from different clinics. Each clinic is staffed by different personnel with different inherent processes and methods of initiating the required steps to receive the medication (prior authorizations, specialty pharmacy referral, and copay assistance programs). This difference in clinics and personnel utilized could contribute to the difference in time to medication receipt with different medications.

Medications with a REMS requirement had a faster time to medication receipt in this study. This was an unexpected and counter-intuitive result. REMS programs have rigorous requirements on who can receive and prescribe the medication. There are also various forms for documentation as well as monitoring requirements before the medication can be dispensed. For these reasons, it was expected that medications with REMS requirements would require more days to medication receipt. One possible explanation for the unexpected result is the familiarity with the REMS requirement processes. As personnel responsible for this documentation become more familiar with the requirements and processes to meet these requirements, it becomes more fluent and less time-consuming and can occur concurrently with the insurance approval process.

The study also found the presence of a secondary insurance program, out-of-pocket cost to patient, use of a patient assistance program, and prior authorization requirements did not significantly affect time to medication receipt. This again was a surprising finding as these are all characteristics that require additional steps or documentation. For medications requiring a prior authorization, it is logical to assume that more time is required to obtain the authorization before referral of the prescription to a specialty pharmacy. Patients with more out-of-pocket costs typically require the use of a copay assistance program. By using a secondary insurance program or a copay assistance program, there is another company that must verify and approve patient financial information (benefits, copays, income, etc.). This would logically increase the amount of steps and time required for the patient to receive the medication. However, we did not find that any of these characteristics had a significant impact on the amount of time required to receive the medication. These results are encouraging and will hopefully prevent patients and prescribers from avoiding the use of these financial assistance methods in fear of delaying the process.

This study had several limitations. First, it was retrospective review of a small number of patients. In addition, there was the potential to exclude some patients with a new prescription, in cases where the physician wrote a prescription without including any ancillary staff members. There was also no breakdown of the time required for each step of the process (prior authorization receipt, specialty pharmacy prescription processing and delivery, etc.), which could be beneficial, as it would provide a more accurate portrayal of the rate-limiting step in the process of obtaining oral antineoplastic agents. Finally, the effects of delayed therapy initiation on cancer outcomes were not evaluated as they were beyond the scope of the study.

## 5. Conclusion

A median of 8 days elapsed between when the prescription for a first-time fill of a new oral antineoplastic agent was written and when it was received by the patient. The medication prescribed, absence of a REMS program, and type of insurance used are factors that increased time to medication receipt. Understanding the median time involved, as well as factors affecting the time to delivery of prescriptions, will help healthcare providers better plan and prepare for the use of oral antineoplastic agents.

## Figures and Tables

**Table 1:** Patient demographics.

Variable	Result number (%) (*N* = 58)
Gender	
Male	32 (55)
Female	26 (45)
Age (years)	
≥60	33 (57)
<60	25 (43)

Median age (years): 60.5	

**Table 2:** Characteristics of 58 patients.

Covariant	Days to receipt			*p*
	*N*	Mean	SD	
Gender				
Male	32	8.28	8.98	0.07
Female	26	10.54	6.33	
Age (year)				
<60	25	9.48	6.92	0.97
≥60	33	9.15	8.70	
Medication prescribed				
Pomalidomide	7	5.86	3.58	0.03
Lenalidomide	6	4.83	2.32	
Temozolomide	11	7.45	3.11	
Capecitabine	16	14.06	12.31	
Others	18	9.00	5.39	
REMS				
Yes	15	6.07	3.56	0.02
No	43	10.42	8.71	
Insurance				
Private	23	10.74	6.28	0.003^*∗*^
Medicaid	8	6.88	5.94	
Medicare AB	11	13.09	13.66	
Medicare D	15	5.67	3.15	
No insurance^*∗*^	1	8.00	n/a	
Secondary insurance				
Yes	9	8.00	3.46	0.94
No	49	9.53	8.49	
Cost to patient				
No costs	31	8.97	9.40	0.22
Some costs	27	9.67	5.92	
Patient assistance program use				
Yes	21	10.43	10.98	0.85
No	37	8.65	5.57	
PA required				
Yes	37	9.70	9.13	0.99
No	21	8.57	5.26	

^*∗*^ *p* value was calculated by excluding one patient without insurance.
